# Prevalence of Anemia, Iron-Deficiency Anemia, and Associated Factors among Children Aged 1–5 Years in the Rural, Malaria-Endemic Setting of Popokabaka, Democratic Republic of Congo: A Cross-Sectional Study

**DOI:** 10.3390/nu13031010

**Published:** 2021-03-21

**Authors:** Branly Kilola Mbunga, Mala Ali Mapatano, Tor A. Strand, Elin Lovise F. Gjengedal, Pierre Zalagile Akilimali, Ingunn Marie S. Engebretsen

**Affiliations:** 1 Kinshasa School of Public Health, University of Kinshasa, Kinshasa, Congo; mapatanow@yahoo.fr (M.A.M.); pierretulanefp@gmail.com (P.Z.A.); 2 Department of Global Public Health and Primary Care, Centre for International Health, University of Bergen, 5009 Bergen, Norway; tor.strand@uib.no (T.A.S.); Ingunn.Engebretsen@uib.no (I.M.S.E.); 3 Faculty of Environmental Sciences and Natural Resource Management, Norwegian University of Life Sciences, 1432 Aas, Norway; elin.gjengedal@nmbu.no

**Keywords:** anemia, iron deficiency, children, malaria, Popokabaka

## Abstract

Iron deficiency (ID), the leading cause of anemia and the most common nutritional deficiency globally, is not well reported among children in malaria-endemic settings, and little is known about its contribution to anemia in these settings. We aimed to assess the prevalence of anemia, the role of ID using multiple parameters, and the factors associated with anemia in a malaria-endemic rural area. We conducted a community-based cross-sectional study of 432 children aged 1–5 years from the Popokabaka Health Zone, Democratic Republic of Congo. Sociodemographic characteristics, medical history, anthropometric parameters, and biochemical parameters were considered. Hemoglobin and malaria prevalence were assessed using rapid finger-prick capillary blood testing in the field. Venous blood samples were analyzed for serum ferritin, serum iron, total iron-binding capacity, and C-reactive protein (CRP) in a laboratory. Anemia was found in 294 out of 432 (68%) patients. Malaria was found in 375 out of 432 (87%), and ID in 1.8% according to diagnosis by adjusted ferritin only and in 12.9% according to transferrin saturation. ID indicators were not significantly correlated with low hemoglobin levels. Malaria, fever, and CRP > 5 mg/L were major factors associated with anemia in Popokabaka. Anemia control should focus on treating inflammatory conditions and infectious diseases among children in such settings.

## 1. Introduction

Anemia, which is characterized by a hemoglobin level below 11.0 g/dL, continues to be a serious global public health problem that particularly affects young children and pregnant women [1]. The World Health Organization (WHO) reported that an estimated 42% of children aged < 5 years are anemic worldwide; the burden is even higher in Africa, reaching 62.3% [2]. According to the most recent Demographic and Health Survey (DHS) and the Multiple Indicators Cluster Survey (MICS), the prevalence of anemia among children aged < 5 years in the Democratic Republic of Congo (DRC) is around 63% [3,4].

While multiple factors are known to cause anemia, the literature [5,6,7,8] shows that iron deficiency (ID), wherein a person has low storage of iron, is the most common nutritional cause [7,8,9]. Over several decades, nutritional interventions have been carried out in most developing countries [7], and nationwide in the DRC [3], to strengthen the prevention of anemia in childhood. One might have expected a decrease in the incidence of anemia, but its prevalence remains high. This is particularly evident in malaria-endemic areas [10]. There is little information to date on ID and its contribution to anemia in the DRC. Large-scale surveys such as the DHS or MICS have not assessed iron parameters, perhaps because of the complex logistics and laboratory analysis required. However, some studies have been conducted, such as the study by Harvey-Leeson et al. in 2016 [11], which reported a discrepancy in ID prevalence (1% when using adjusted ferritin and 58% with serum transferrin) among children aged 2–5 years old in the Kongo Central Province, DRC. The study found that ID anemia (IDA) never exceeded 20%, regardless of the iron biomarker used. Bahizire et al. [12] conducted a study in the South Kivu Province, DRC, and reported an ID prevalence of 10.4% (using inflammation-adjusted ferritin), observing that ID contributed to less than a fifth of anemia cases in children aged < 5 years.

However, according to the WHO [13], half of the anemia cases are expected to be explained by ID. In this study, we aimed to assess the prevalence of anemia and the role of ID among children aged <5 years who presented with anemia, to suggest specific directions for useful anemia-control strategies. We also assessed possible risk and protective factors associated with anemia.

## 2. Materials and Methods

### 2.1. Study Design and Setting

We conducted a community-based cross-sectional study in the Popokabaka Health Zone, DRC, between May and June 2019. This region is an entirely rural area located in Kwango Province, where the population is facing food insecurity and poverty [3]. The health system is organized around the referral hospital and health centers, coordinated by the Health Zone management. National strategies regarding malaria and anemia control are also implemented. For instance, iron and folic acid supplementation to women during pregnancy and Infant and Young Child Feeding (IYCF) support for children aged up to 2 years are organized at the facility and community levels [3,4]. Malaria-control strategies are integrated with other disease-control programs under Health Zone management [4]. These include community screening and treatment for mild malaria by community health workers, the integrated management of severe cases at health facilities, epidemic management, selective vector control, periodical distribution, and campaigning for the use of insecticide-treated bed nets. *Plasmodium falciparum* is the most prevalent malaria-causing species found in DRC [4]. Available information for the Kwango province shows that stunting (43%) and wasting (8%) are prevalent in children [4]. The diversity of food is limited, and the soil is sandy and arid, not permitting the growth of a wide variety of crops. Farmers preferably grow bitter cassava as a staple food because of its resistance to drought and the energy value of its roots. Animal food sources, dairy products, and sea products are seldom consumed.

### 2.2. Participants and Sampling

A total of 432 children aged 12–59 months were included in this study, based on a proportional sample size calculation for anemia prevalence = 0.59, a precision d of 0.075, a design effect of two, and an expected response rate of 0.80. Children were selected using a three-stage cluster sampling technique. First, we selected five clusters (health areas) among the nine accessible areas through a probability-proportion-to-size technique. The term “accessible” considered the time constraints for collecting venous blood samples from the field and being able to process them within 3 h at the Popokabaka Hospital. Only 9 of the 25 health areas fitted this technical constraint. Second, we randomly selected 3 villages in each cluster. In the last stage, an equal number of 30 households having children aged 12–59 months old were systematically selected from a detailed list pre-established by community workers in each village. Only one child from each household was selected and assessed. A parent had to provide written consent for their child to participate in the study. Children for whom the parents refused blood drawing or children who were hospitalized for any diseases in the two previous weeks were excluded.

### 2.3. Data Collection and Procedures

Data were collected using a questionnaire completed on tablet computers using the Survey CTO collect application. The questionnaire consisted of eight modules: household characteristics; water, hygiene, and sanitation (WASH); household food security (Household Food Insecurity Access Scale-HFIAS); child health history; IYCF; anthropometric measures; dietary practices (24 h recall and food frequency); and biochemical sampling.

Data collection took two consecutive days in each cluster, three teams working in parallel for each of the three selected villages of the cluster. A team comprising three data collectors and two phlebotomists was formed. On the first day, the data collectors visited the households, obtained consent forms, and completed the surveys for each child selected in the village. The biological mother or caretaker responded to the questionnaire while anthropometric measurements were taken for the child. When finished, the personnel provided a card to the mother with the correct personal identifier for blood collection the next day. On the second day, the phlebotomists went to the same villages at a specific identified location (the Health Center or another appropriate place) from 7 to 9 am for blood collection and hemoglobin and malaria rapid testing. The mothers were then invited to bring the children and the cards for blood collection.

The data collectors were trained by the investigator on the survey questionnaire and interview techniques, and the phlebotomists were trained by a lab expert on appropriate standard operating procedures (SOPs) and blood sample management.

### 2.4. Blood Processing and Management

The phlebotomist first performed a capillary finger-prick test for hemoglobin (Hgb) assessment (Hemocue 301) and a rapid test for malaria in the field. The information was recorded using tablet computers and the Survey CTO application. Additionally, the phlebotomist collected up to 6 mL of venous blood from the child using trace-element-free serum BD vacutainers (BD-368380) and powder-free sterile disposable gloves. Tourniquet application took a maximum of one minute. The collected blood was allowed to clot for at least 30 min in the field at room temperature and was then transported to the Popokabaka Hospital within 3 h. There, it was centrifuged, and the supernatant was separated at 2300 rpm for 10 min using a Hettich Rotor 32A centrifuge (Tuttlingen, Germany). The serum was aliquoted into two polypropylene vials: 0.5 mL tricoded FluidX, Brooks Life Science vials and 2 mL Sarstedt vials.

All the vials were immediately stored in a −40 °C freezer that worked nonstop on solar energy during the day and on a generator overnight in Popokabaka. When the survey was complete, the samples were transported from Popokabaka to Kinshasa (a 12 h vehicle trip with self-transported fuel) stored in liquid nitrogen. Then, every sample was stored at −80 °C in an ultra-low freezer at the Kinshasa School of Public Health for a week before being shipped on dry ice to Norway.

All the 0.5 mL vials were sent to Haukeland University Hospital (Bergen, Norway) for the analysis of serum ferritin (using electrochemiluminescence immunoassay (ECLIA)), C-reactive protein (S-CRP) (using the immunoturbimetry method), and total iron-binding capacity (S-TIBC, using the Berekna equation calculation: s-TIBC = s-Transferrin * 25.1). Two-milliliter vials were sent to the Norwegian University of Life Sciences (Ås, Norway) for the analysis of serum iron and other minerals (using the Agilent 8900 Triple Quadrupole inductively coupled plasma mass spectrometer (ICP-MS)).

Transferrin saturation (TSAT), expressed as a percentage, was then calculated as the value of serum iron divided by the TIBC.

### 2.5. Transformation of Variables 

Anthropometric indices including the weight for height, height for age, weight for age, and mid-upper-arm circumference for age and their Z-scores were calculated using the WHO Anthro software. Wasting was defined as a weight-for-height Z-score (WHZ) < −2, stunting was defined as a height-for-age Z-score < −2, and underweight was defined as a weight-for-age Z-score < −2. Wealth index quintiles were generated using principal component analysis on ownership variables in the household. The food consumption score was calculated using data from the Food Frequency Questionnaire (FFQ) and categorized as adequate, borderline, and inadequate consumption. The dietary diversity variable was generated from 24 h food recall, capturing the intake of common food items in the area. The Household Food Insecurity Access Score (HFIAS) and its categories were also calculated.

### 2.6. Anemia and ID Definition

Anemia was defined as Hgb levels < 11 g/dL [14]. According to the level, it was classified as mild, moderate, or severe when the Hgb concentrations were 10–10.9, 7.0–9.9, or <7.0 g/dL, respectively. ID was defined as serum ferritin concentrations < 12 µg/L in the absence of inflammation [14]. To account for inflammation [8], we used the regression-correction approach developed by the Biomarkers Reflecting Inflammation and Nutritional Determinants of Anemia (BRINDA) [15] to estimate the prevalence of ID. The regression-correction approach followed a three-step process. First, we defined internal reference values for inflammatory markers (CRPref) as the tenth percentile [15]. Then, we estimated the regression coefficient (β) for the association between CRP and ferritin using univariable linear regression models, with ferritin as the dependent variable. Finally, we calculated CRP-adjusted ferritin values using the following equation:

*Adjusted ferritin = unadjusted ferritin − β (CRPobs − CRPref).*

We also estimated ID by calculating TSAT by dividing the serum iron (Fe^3+^) levels by the TIBC. A child is said to have ID when TSAT is <20% [14].

A child was said to have malaria when there was a positive result in the rapid malaria test for *Plasmodium falciparum*.

### 2.7. Statistical Analysis

All the data were merged and analyzed using STATA 14.0. We performed a descriptive analysis. The median and 25th–75th percentiles are reported for continuous variables that were not normally distributed, while the mean (SD) is used for continuous variables that were normally distributed. Frequencies are used to describe categorical data. The confidence intervals (CIs) for prevalence were calculated using a normal Z-test. Moreover, an independent-sample t-test or the Wilcoxon rank-sum test were used to compare, respectively, mean or median values. A Pearson chi-squared test was used to test for association, with anemia as the outcome. The Spearman correlation coefficients were used to describe the relationships between the iron biomarkers and hemoglobin concentrations. Linear regression was used to adjust ferritin for inflammation (CRP). Bivariable and multiple logistic regression were used to identify factors associated with anemia, and the crude and adjusted odds ratios (ORs) were reported. The regression model was constructed by a forward stepwise selection approach with covariate inclusion probability (*p*-value of crude OR) < 0.20. Multicollinearity was checked for all the covariates included in the regression model. All the statistical analyses were performed at the 0.05 level of confidence.

## 3. Results

### 3.1. Characteristics of the Study Population

In total, 432 children aged 1–5 years old were included in this study. The general characteristics of the enrolled children are shown in Table A1. The boy/girl ratio was 1:1, and their median (P25–P75) age was 32 (22–43) months. Children aged 48–59 months were underrepresented compared to those in the other age groups. Fever onset during the 2 weeks preceding the study visits was common among the children in Popokabaka and more so among anemic children (63.3%) than those without anemia (45.7%) (Table A2). The results also support the existence of childhood health and anemia-control strategies implemented in Popokabaka, but not with optimal coverage (Table A2). Almost seven out of ten children had benefitted from deworming in the previous 6 months, while half had received vitamin A in the previous six months. About one third (35.6%) were treated with iron supplements or syrups, suggesting that nutritional therapy for anemia was also practiced. 

Stunting was the most common form of malnutrition, with more than half of the children (56%) affected, and every tenth (11%) child exhibited wasting. Only one in three had a diversified diet, and approximately 3% of the children were defined as being obese.

### 3.2. Prevalence and Distribution of Anemia and Iron Deficiency

The prevalence of malaria in Popokabaka was 86.8% (95% CI, 83.6–90.0%) according to *Plasmodium falciparum* rapid testing. We found that anemia was also common, at 68.1% (95% CI, 64.0–72.0%), with severe anemia seen among 3.3% (95% CI, 1.9–5.4%) of the children. 

Half of the children (49.4%) had signs of inflammation, with CRP > 5 mg/L. This proportion was significantly higher among anemic children than nonanemic children (53.5% vs. 40.7%, *p* value = 0.001).

However, ID was less prevalent when taking all the iron biomarkers into account. First, considering that ferritin increases in the acute phase of inflammation, we determined the unadjusted ferritin prevalence by excluding the ferritin values of children with CRP > 5 mg/L during calculation. Only one out of 193 children presented with ID (ferritin <12 µg/L). In another approach, accounting for inflammation as proposed by the BRINDA project [15], we found an ID prevalence of 1.8% (95% CI, 0.5–3.0%) using the ferritin biomarker adjusted for CRP. The latter approach, where the adjustment was performed using linear regression, allowed the inclusion of all the observations where we had measured serum CRP and ferritin concentrations. This prevalence was lower than those based on other indicators. Table A3 shows that the ID prevalence according to the TSAT biomarker was the highest, at 12.9% (95% CI, 9.6–16.1%).

We estimated the IDA prevalence and the contribution of ID to anemia, and the former 7.5% according to transferrin saturation and 1.5% according to adjusted ferritin. Thus, the remaining anemia cases were not iron-deficiency-related.

### 3.3. Correlation with Iron Biomarkers 

Hgb was not correlated with transferrin saturation (TSAT) but showed a weak, negative correlation with ferritin (r = −0.17) and serum iron (r = 0.07) (Table A4). In addition, Hgb was negatively correlated with CRP, suggesting a possible role of inflammatory conditions or diseases in anemia genesis.

### 3.4. Association with Anemia

Table A5 shows the multivariable regression analysis with determinants for anemia among children aged under five years in Popokabaka. We found that malaria (OR, 4.08 (2.18–9.68)), fever during the previous two weeks (OR, 1.71 (1.08–2.70)), and signs of inflammation (CRP > 5 mg/L) (OR, 1.65 (1.05–2.59)) were associated with anemia. However, ID according to TSAT was inversely associated with anemia (OR, 0.50 (0.27–0.97)), meaning that the presence of ID did not imply or protect against anemia for the data collected. 

## 4. Discussion

In the present study, we demonstrated the burden of ID and anemia using multiple biomarkers for a representative community-based sample of children in a poor part of the DRC, which, to the best of our knowledge, had not been assessed before. Our study found that anemia was highly prevalent (68.1%) among children aged < 5 years, while ID was remarkably low (12.9% according to TSAT, 7.9% according to TIBC, and 1.8% according to regression-adjusted ferritin biomarkers). This anemia prevalence seemed much higher than the previously reported national prevalence (59.9%, *n* = 8280) according to the latest Demographic and Health Survey in 2014 [3]. This may indicate that children in Popokabaka are at the highest risk of anemia complications in the DR Congo. Harvey-Leeson et al. [11] reported an anemia prevalence rate of 44% in Kongo Central Province, while Bahizire [12] reported a prevalence of 46.6% in rural Kivu, both of which are areas known for long periods of civil unrest. This difference could be explained by the fact that our study was specifically carried out in a small geographical area, a “health zone”, where the problem is indeed serious. In this case, the prevalence may be higher than that in a much larger research area. Another reason of this difference is that we did not adjust the Hgb levels for altitude and ethnicity, and this may have increased the false-negative proportion in our anemia classification. In other words, a reduction in this prevalence could be expected if those factors were also considered.

The WHO reports that almost 50% of anemia could be caused by ID and argues that ID is the “single” largest contributor to the anemia burden [16]. However, this was not the case in our study population. We found a very low prevalence of ID, with the highest prevalence being 12.9% according to TSAT. This is consistent with the findings of Harvey-Leeson [11], in the western part of the DRC (Kongo Central Province), and Bahazire [12], in the eastern part of the DRC (Kivu Province). Gebreegziabher et al. [17] reported the same situation in rural southern Ethiopia among women of reproductive age. By contrast, Muriuki et al. investigated iron prevalence across five countries in Africa [18] and found a much higher pooled prevalence of ID, 34.3%. The difference might be due to the high prevalence of malaria and inflammation in our population. In fact, under these conditions, the prevalence of ID according to ferritin could be underestimated because of the bias of ferritin toward higher concentrations. Another reason might be the systematic iron supplementation strategy for children with anemia in Popokabaka, causing iron repletion. Motadi et al. [19] also found a much higher prevalence according to TSAT (12%) than adjusted ferritin (2.3%). Moreover, Muriuki et al. [18] reported that TSAT is the most sensitive and specific marker compared to the gold standard, which is regression with adjusted ferritin in African children. Dignass et al. [20] also suggested the use of TSAT rather than ferritin in the context of chronic inflammatory diseases. In a review published in 2020, Capellini et al. [21] emphasized that, in the context of inflammation, the TSAT biomarker is more sensitive and less biased than a ferritin cutoff of 100 µg/L. 

The high anemia prevalence with very low iron storage depletion in this highly prevalent malaria context cannot be explained in the present study. However, we anticipate, based on the existing literature [22,23], that in the malaria-endemic context, hemoglobin genetic disorders (e.g., the sickle cell trait) and high iron supplementation may be common explanations. The biological pathway involves an increase in iron absorption capacity in some of these conditions even though the storage capacity is normal. Taking into account the low consumption of animal-source foods in Popokabaka, a possible explanation is a frequent consumption of iron-rich vegetables (e.g., cassava and amaranth) [24] and the widespread self-prescription of iron tablets [12]. 

Another explanation could be possible iron supplementation (syrups or tablets) administered at home to anemic children by parents in Popokabaka, even though only 35% of those who took part in our study reported that their children had received iron supplementation in the previous three months (Table A1).

In a recent review, Camaschella et al. [25] explained the endocrine regulatory role of hepcidin in iron balance. According to their review, proinflammatory cytokines such as interleukin-6 increase hepcidin levels during acute and chronic inflammation, which leads to iron-restricted erythropoiesis and anemia of inflammation. According to this pathway, we would have expected a high prevalence of iron deficiency in children living in a malaria-endemic setting. However, this was not the case in our study. We found a low prevalence of ID in the children of Popokabaka. Camaschella et al. discussed another pathway through which unexpectedly elevated levels of serum iron might be induced along with anemia, especially in individuals with iron-loading anemias or chronic anemia: low levels of, the inhibition of, or the loss of control of hepcidin. We did not assess hepatic hepcidin in our study but suspect that this protein would be low and probably impacted by recurrent protein malnutrition (in our study, we found that one in ten children had acute malnutrition while one in two was chronically malnourished) or other hepcidin disorders.

We estimated the association between anemia and other factors in a multivariate logistic regression analysis, and we found that fever in the previous two weeks, malaria status, and inflammation status (CRP > 5 mg/L) were factors associated with anemia. Taking into account the weak correlation of hemoglobin with iron biomarkers, we hypothesized from these results that the remaining cases of anemia could be explained or have been caused by malaria or other diseases that we did not explore in our study. Unfortunately, we did not assess other illnesses such as helminthic infections [26,27]. Chaparro and Suchdev [28], when framing causal models for anemia determinants, emphasized malaria, poor sanitation, underweight, inflammation, stunting and vitamin A deficiency.

We used rapid malaria tests to determine the prevalence of parasitemia based only on the presence of *Plasmodium falciparum*. This could have underestimated the true prevalence of malaria in terms of parasite count. In a review of how malaria was associated with anemia, White [10] stated that, in endemic malaria contexts, young children had repeated symptomatic infections, with high rates of asymptomatic parasitemia, and often had chronic anemia.

In our study, anemia prevalence was indifferently distributed within nutritional parameters—food insecurity, dietary diversity, and anthropometric indicators—which did not explain any of the variation in anemia. This finding is in contrast to the summary findings reported by the WHO [7], indicating that multiple nutrient deficiencies of both minerals and vitamins are causes of anemia.

The present study had several strengths. This was a community-based biochemical study for a hard-to-reach rural settlement with many sample-conservation constraints. We obtained a representative study population from Popokabaka through a probabilistic sampling scheme with a large sample size. We demonstrated that iron deficiency might not be the main cause of anemia in such malaria-endemic areas, that malaria itself or other inflammatory conditions may be leading factors. We examined multivariate regression models and checked for multicollinearity to validate the associated factors found.

However, the study had several limitations. The study population was restricted to technically accessible areas in the study area, as explained in Section 2.2, to avoid damage to proteins and possible hemolysis due to long distances from the field to the referral hospital. Future research in such hard-to-reach areas could consider on-site blood processing and serum conservation for a higher representation of distant areas. We acknowledge that the analysis of more biomarkers would have had scientific value, such as soluble transferrin receptors and hepcidin. We conducted a cross-sectional study, so we cannot argue any causality for the relationships reported in this study. We think that the protective effect of iron deficiency on anemia was related to the medium sample size, to such an extent that this would become null with a bigger sample. 

Despite the fact that the interpretation of Hgb concentrations requires some adjustments (for altitude and ethnicity) as recommended by the literature [29,30], for this study, we did not consider any adjustments for altitude or ethnicity. The reasons were that we did not capture geo coordinates (altitude) during data collection and that the Popokabaka Health Zone is a small, relatively flat and low-altitude region inhabited by the same Yaka population. In these conditions, the misclassification of anemia would have been minor and would not have affected our findings.

Finally, anemia has complex pathways, and there are likely to be other important factors involved that were not measured.

## 5. Conclusions

Iron deficiency should be considered prevalent in populations when its prevalence is more than 20%. In this study, we demonstrated a low iron deficiency prevalence within a context of high anemia and malaria prevalence in Popokabaka. ID was not a risk factor for anemia in this study. However, malaria was a major contributor to anemia. Further research with more hematological biomarkers is needed to characterize this anemia, which was not explained by ID. Due to the findings from this study, we suggest that anemia control strategies in Popokababa focus on malaria prevention and other childhood infectious-disease controls for effective impact. 

## Data Availability

The dataset of this study can be made available on reasonable request to B.K.M.

## Appendix A

**Table A1:** General characteristics and anthropometry of children aged 1–5 years in Popokabaka.

	Total	Anemia		*p*-Value
Report Unit		Yes	No	
**Age (months)** Median (P25–P75)	32 (22–43)	31 (22–42)	33 (23–45)	0.869
**Age Groups (months)** *n* (%)				0.985
12–23	124 (28.7)	86 (29.3)	38 (27.5)	
24–35	120 (27.8)	81 (27.5)	39 (28.3)	
36–47	116 (26.8)	78 (26.5)	38 (27.5)	
48–59	72 (16.7)	49 (16.7)	23 (16.7)	
**Gender** *n* (%)				0.927
Boys	224 (51.8)	152 (51.7)	72 (52.2)	
Girls	208 (48.2)	142 (48.3)	66 (47.8)	
**Height-for-Age Z-score** Mean (SD)	−2.2 (1.7)	−2.2 (1.7)	−2.0 (1.7)	0.255
**Weight-for-Height Z-score** Mean (SD)	−0.6 (1.3)	−0.7 (1.2)	−0.3 (1.4)	0.011
**Weight-for-Age Z-score** Mean (SD)	−1.5 (1.4)	−1.7 (1.3)	−1.3 (1.3)	0.014
**Stunting** *n* (%)				0.632
Yes	242 (56.0)	167 (56.8)	75 (54.4)	
No	190 (44.0)	127 (43.2)	63 (45.6)	
**Wasting** *n* (%)				0.913
Yes	48 (11.1)	33 (11.2)	15(10.9)	
No	384 (88.9)	261 (88.8)	123 (89.1)	
**Underweight** *n* (%)				0.103
Yes	152 (35.2)	111 (37.8)	41 (29.7)	
No	280 (64.8)	183 (62.2)	97 (70.3)	
**Diversified diet in last 24 h** *n* (%)				0.432
Yes	133 (39.8)	87 (29.6)	46 (33.3)	
No	299 (69.2)	207 (70.4)	92 (66.7)	

**Table A2:** Clinical characteristics of children aged 1–5 years in Popokabaka.

	Total	Anemia		*p*-Value
		Yes	No	
**Diarrhea in last 2 weeks** *n* (%)				0.187
Yes	78 (18.1)	58 (19.7)	20 (14.5)	
No	354 (81.9)	236 (80.3)	118 (85.5)	
**Bloody stools in last 2 weeks**				0.801
Yes	18 (4.17)	13 (4.4)	5 (3.6)	
No	414 (95.8)	281 (95.6)	133 (96.4)	
**Fever in last 2 weeks**				0.001
Yes	249 (57.6)	186 (63.3)	63 (45.7)	
No	183 (42.4)	108 (36.7)	75 (54.4)	
**Cough in last 2 weeks**				0.819
Yes	141 (32.64)	97 (33.0)	44 (31.9)	
No	291 (67.4)	197 (67.0)	94 (68.1)	
**Zinc tablets in last 2 weeks**				0.649
Yes	23 (5.3)	17 (5.8)	6 (4.4)	
No	409 (94.7)	277 (94.2)	132 (95.6)	
**Vitamin A supplements in last 6 months**				0.520
Yes	232 (53.7)	161 (54.7)	71 (51.5)	
No	200 (46.3)	133 (45.2)	67 (48.5)	
**Deworming in last 6 months**				0.865
Yes	306 (70.8)	209 (71.1)	97 (70.3)	
No	126 (29.2)	85 (28.9)	41 (29.7)	
**Iron supplements in last 3 months**				0.077
Yes	154 (35.6)	113 (38.4)	41 (29.7)	
No	278 (64.4)	181 (61.6)	97 (70.3)	
**Sleeping under mosquito nets**				0.406
Yes	260 (60.2)	173 (58.8)	87 (63.0)	
No	172 (39.8)	121 (41.2)	51 (37.0)	

**Table A3:** Prevalence and distribution of anemia, iron deficiency, and malaria.

	*n*	%	(95% CI )
**Anemia**			
Anemia, all types (Hgb < 11.0 g/dL)	294/432	68.1	(64.0–72.0)
Severe anemia (Hgb < 7.0 g/dL)	14/432	3.3	(1.9–5.4)
Moderate anemia (Hgb 7.0–9.9 g/dL)	153/432	35.4	(31.0–40.1)
Mild anemia (Hgb 10.0–10.9 g/dL)	127/432	29.4	(25.3–33.9)
**Iron Deficiency**			
ID as unadjusted ferritin < 12 µg/L when CRP < 5	1/193	0.5	(0.5–1.5)
ID as CRP-adjusted ferritin < 12 µg/L	7/400	1.8	(0.5–3.0)
ID as TIBC > 66 umol/L	33/419	7.9	(5.2–10.5)
ID as TSAT < 20%	55/412	12.9	(9.6–16.1)
**Malaria and Inflammation**			
Malaria (positive rapid *P. falciparum* test)	375/432	86.8	(83.6–90.0)
CRP < 5 mg/L	207/419	49.4	(44.6–54.0)

**Table A4:** Spearman correlation of hemoglobin with iron biomarkers, r coefficient (*p*-value).

	Hemoglobin	Ferritin	CRP	TSAT	Serum Iron	TIBC
**Hemoglobin**	1.000					
**Ferritin**	−0.17 (0.002)	1.000				
**CRP**	−0.19 (0.000)	0.35 (0.000)	1.000			
**TSAT**	−0.02 (0.765)	0.31 (0.000)	−0.08 (0.161)	1.000		
**Serum Iron**	0.07 (0.170)	0.07 (0.170)	−0.18 (0.000)	0.83 (0.000)	1.000	
**TIBC**	0.14 (0.011)	−0.51 (0.000)	−0.19 (0.000)	−0.46 (0.000)	0.04 (0.502)	1.000

**Table A5:** Factors associated with anemia, crude and adjusted odds ratios (ORs) reported.

	Bivariate Estimates			Multivariate Logistic Regression Analysis		
	OR _Crude_	CI	*p* Value	OR _Adjusted_	CI	*p* Value
**Age**	0.99	0.97–1.01	0.505	-	-	-
**Household size**	1.05	0.96–1.13	0.277	-	-	-
**Sex**						
Girl	1.01	0.68–1.52	0.927	-	-	-
Boy	1					
**Diarrhea in last 2 weeks**						
Yes	1.45	0.83–2.52	0.187	1.26	0.63–2.52	0.519
No	1					
**Bloody stools in last 2 weeks**						
Yes	1.23	0.43–3.52	0.699	-	-	-
No	1					
**Fever in last 2 weeks**						
Yes	2.05	1.36–3.09	0.001	1.71	1.08–2.70	0.020
No	1					
**Deworming in last 6 months**						
Yes	1.03	0.66–1.61	0.865	-	-	-
No	1					
**Iron supplementation last 3 months**						
Yes	1.48	0.96–2.28	0.077	1.49	0.92–2.40	0.102
No	1					
**Multiple micronutrients in last 3 months**						
Yes	0.86	0.51–1.44	0.576	-	-	-
No	1					
**Sleeping under mosquito nets**						
Yes	0.83	0.55–1.27	0.406	-	-	-
No	1					
**Stunting**						
Yes	1.10	0.74–1.65	0.632	-	-	-
No	1					
**Wasting**						
Yes	1.04	0.54–1.97	0.913	-	-	-
No	1					
**Underweight**						
Yes	1.43	0.93–2.21	0.103	1.37	0.73–2.54	0.321
No	1					
**Dietary diversity**						
Yes	0.84	0.54–1.30	0.432	-	-	-
No	1					
**CRP**						
>5 mg/L	1.68	1.10–2.54	0.015	1.65	1.05–2.59	0.029
≤5 mg/L	1					
**ID by TSAT**						
Yes	0.62	0.34–1.12	0.116	0.50	0.27–0.97	0.038
No	1					
**Malaria**						
Yes	5.02	2.78–9.05	<0.001	4.08	2.18–9.68	<0.001
No	1					
